# 

**Published:** 1891-05

**Authors:** 


CONTENTS:

PAGE

ORIGINAL ARTICLES:
- Uniform Veterinary Education. By W. Horace Hoskins, D. V. S.... 209
- Diverticulum of the Œsophagus. By A. W. Clement, V. S.......... 216
- Lymphatics. By B. F. King, V. S.......... 218
- Cannabis Indica in Colic. By S. Stewart, M. D., D. V. M.......... 222
- Tuberculosis in Tigress.......... 225
- Age of the Horse, Ox, Dog, and Other Domesticated Animals. By R. S. Huidekoper, M. D., Vet.......... 226
- Recent Patents.......... 232

EDITORIAL:
- University of Nebraska.......... 240
- Pleura-pneumonia Contagiosa.......... 241

SELECTIONS FROM FOREIGN JOURNALS:
- German Review.......... 243
- Spanish Review.......... 247

SOCIETY PROCEEDINGS:
- Massachusetts Veterinary Association.......... 248
- Veterinary Medical Association of New Jersey.......... 252
- Keystone Veterinary Medical Association.......... 253, 254, 257

VETERINARY COLLEGES:
- New York College of Veterinary Surgeons; Commencement.......... 258
- Montreal Veterinary College; Commencement.......... 260
- Ontario Veterinary College; Commencement.......... 261

REVIEW DEPARTMENT:
- Books and Pamphlets Received.......... 264

